# Effects of Monoamino-Oxidase-A (MAO-A) Inhibition on Skeletal Muscle Inflammation and Wasting through Pancreatic Ductal Adenocarcinoma in Triple Transgenic Mice

**DOI:** 10.3390/biomedicines11030912

**Published:** 2023-03-15

**Authors:** Simon K. P. Schmich, Jan Keck, Gabriel A. Bonaterra, Mirjam Bertoune, Anna Adam, Beate Wilhelm, Emily P. Slater, Hans Schwarzbach, Volker Fendrich, Ralf Kinscherf, Wulf Hildebrandt

**Affiliations:** 1 Anatomy and Cell Biology, Department of Medical Cell Biology, University of Marburg, 35032 Marburg, Germany; 2 Department of Visceral-, Thoracic- and Vascular Surgery, Philipps University Marburg, 35043 Marburg, Germany

**Keywords:** cachexia, pancreatic cancer, KPC model, skeletal muscle atrophy, E3-ligases, cytokines, harmine

## Abstract

Cancer cachexia describes a syndrome of muscle wasting and lipolysis that is still largely untreatable and negatively impacts prognosis, mobility, and healthcare costs. Since upregulation of skeletal muscle monoamine-oxidase-A (MAO-A), a source of reactive oxygen species, may contribute to cachexia, we investigated the effects of the MAO-inhibitor harmine-hydrochloride (HH, intraperitoneal, 8 weeks) on muscle wasting in a triple-transgenic mouse model of pancreatic ductal adenocarcinoma (PDAC) and wild type (WT) mice. Gastrocnemius and soleus muscle cryo-cross-sections were analyzed for fiber type-specific cross-sectional area (CSA), fraction and capillarization using ATPase- and lectin-stainings. Transcripts of pro-apoptotic, -atrophic, and -inflammatory signals were determined by RT-qPCR. Furthermore, we evaluated the integrity of neuromuscular junction (NMJ, pre-/post-synaptic co-staining) and mitochondrial ultrastructure (transmission electron microscopy). MAO-A expression in gastrocnemius muscle was increased with PDAC vs. WT (immunohistochemistry: *p* < 0.05; Western blot: by trend). PDAC expectedly reduced fiber CSA and upregulated IL-1β in both calf muscles, while MuRF1 expression increased in soleus muscle only. Although IL-1β decreased, HH caused an additional 38.65% (*p* < 0.001) decrease in gastrocnemius muscle (IIBX) fiber CSA. Moreover, soleus muscle CSA remained unchanged despite the downregulation of E3-ligases FBXO32 (*p* < 0.05) and MuRF1 (*p* < 0.01) through HH. Notably, HH significantly decreased the post-synaptic NMJ area (quadriceps muscle) and glutathione levels (gastrocnemius muscle), thereby increasing mitochondrial damage and centronucleation in soleus and gastrocnemius type IIBX fibers. Moreover, although pro-atrophic/-inflammatory signals are reversed, HH unfortunately fails to stop and rather promotes PDAC-related muscle wasting, possibly via denervation or mitochondrial damage. These differential adverse vs. therapeutic effects warrant studies regarding dose-dependent benefits and risks with consideration of other targets of HH, such as the dual-specificity tyrosine phosphorylation regulated kinases 1A and B (DYRK1A/B).

## 1. Introduction

Cachexia describes a complex inflammatory, catabolic syndrome that is highly prevalent in chronic diseases, such as cancer and manifests itself by wasting of skeletal muscle with or without loss of fat mass [1,2], both of which cannot be compensated for by food intake [3]. Cachexia has a massively negative impact on the patients’ quality of life [1,4,5] and imposes high costs on healthcare systems [6,7]. Despite multiple therapeutic approaches, including nutrition, exercise, anti-inflammatory drugs, such as NSAID, megestrol, or cannabis [8,9,10,11,12,13], a causal treatment does not exist until now.

Although cachexia is a multi-organ syndrome [14], the role of skeletal muscle tissue during its development is crucial. Cachectic patients show a dramatic decrease in life expectancy [15], which is aggravated by the occurrence of obesity and occult muscle loss [16,17]. On a histological level, a wide range of human and animal studies, including pancreatic, lung, and colon cancer, show that a loss of fiber types I and II cross-sectional area (CSA) is the main feature of muscle wasting, in addition to altered myofibrillar structure, local apoptosis, or impaired regeneration, and, in some cases, fiber-type transition [4,18,19,20,21,22,23]. Thereby, fiber atrophy is found in comparison to both healthy controls and non-cachectic tumor-bearing animals or humans. This phenotype varies widely between muscle and fiber type, the entity, mass, or inoculation site of tumor, and the definition and stage of cachexia, among other factors [18,24,25,26,27,28,29].

Oxidative stress is considered to be a major factor of muscle wasting [30,31]. In glucocorticoid-induced proteolysis, gene expression of monoamine oxidase A (MAO-A) is upregulated in skeletal muscle, and thus leads to an increased production of H_2_O_2_ and related reactive oxygen species (ROS) [32]. In several studies on cardiac and skeletal muscle, ROS have been shown to induce mitochondrial damages, apoptosis, fibrosis, etc. [33,34,35,36,37].

Since to date, the impact of MAO-derived ROS on cancer cachexia has remained unclear, we presently aimed to evaluate for the first time the expression of MAO-A protein and the effect of MAO-A inhibition in cancer-related muscle wasting. Since PDAC is clinically associated with the highest incidence and most rapid progression of cachexia as a major contributor to this 4th and 5th leading cause of cancer-associated death [14,38,39,40,41,42,43], we chose a mouse model of orthotopic PDAC established by Hingurani et al. [44]. It is based on the 3x-transgenig Kras^G12D/+^; LSL-Trp53^R172H/+^; Pdx-1-Cre (KPC) mutants (*Kras:* Kirsten Rat Sarcoma, Trp53: Transformation-related protein 53, *Pdx-1-Cre:* Pancreatic and duodenal homeobox 1-cyclization recombination) and commonly utilized for PDAC research and preclinical therapeutic testing, since it mimics well clinical features of human PDAC, such as metastatic pattern (liver 80%, lung 50–60%, adrenal gland 20%, and peritoneum 20–30%)*,* ascites and, importantly, of cachexia development [44,45,46]. Moreover, it recapitulates the mutant-based cancerogenesis from pancreatic intraepithelial neoplasia (PanIN) stages 1–3 to PDAC [44,45,47,48], with a PDAC incidence of 96.3% [44]. According to a previous study of our group, this KPC model develops a likely inflammation-driven fiber atrophy in both, ‘red’ (soleus) and ‘white’ (gastrocnemius) muscle [49]. This muscle wasting coincides with the completion of cancerogenesis and occurs in the absence of altered integrity of mitochondrial ultrastructure or neuromuscular junction (NMJ).

Using this KPC mouse model, we presently evaluated the effect of MAO inhibition via the β-carboline alkaloid harmine-hydrochloride (HH), a competitive and selective MAO-A inhibitor [50,51,52] on cancer-related muscle wasting, with regard to histomorphometry of fiber types, pro-inflammatory, -atrophic, and -apoptotic gene expression, capillarization, NMJ, intramyocellular glutathione (GSH) content and redox state, and relevant transcripts. In addition, the effect of this therapeutic approach was assessed in WT mice without cachexia. The study focused on the hindlimb muscles as commonly performed in the field of cancer- or other disease-related cachexia [4,18,31,49] and evaluated the mixed slow-twitch (‘red’) soleus muscle (containing fiber types I, IIA, and IIBX) as well as the fast-twitch (‘white’) gastrocnemius or quadriceps muscle, which predominantly consists of type IIBX fibers.

## 2. Material and Methods

### 2.1. Tumor Mouse Model

**Figure 1:**
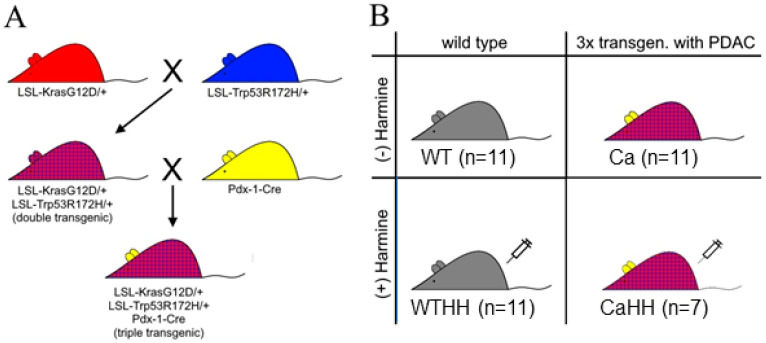
Scheme of the genetic mouse cross-breeding (*LSL: Lox-Stop-Lox*, *Kras*: Kirsten Rat Sarcoma, G12D/+: Glycine/Aspartate substitution in Codon 12, Trp53: Transformation-related protein 53, R172H/+: Arginine/Histidine substitution in Codon 172, *Pdx-1-Cre:* Pancreatic and duodenal homeobox 1-cyclization recombination) [45] (**A**), and the four different groups under test, i.e., wild type mice with (WTHH) or without (WT) HH treatment as well as triple transgenic PDAC-bearing mice with (CaHH) or without (Ca) HH treatment (**B**).

The present study used a triple transgenic mouse model of PDAC first described by Hingorani et al. [45]. It is based on three mutations—Trp53, Kras, and Pdx-1-Cre (Figure 1)—and largely recapitulates the development of PDAC in humans [45]. Mice were cross-bred by the Biomedical Research Centre of the University of Marburg and received food and water ad libitum with a periodic day-night cycle of 12 h. At the age of 3 months, each group of triple transgenic and wild type (WT) mice was treated intraperitoneally with HH (30 mg/kg/day) for 2 months.

After the treatment, the mice were sacrificed by cervical dislocation and the right triceps surae (i.e., gastrocnemius, soleus, and plantaris muscle) as well as the quadriceps muscle were removed and snap-frozen in liquid nitrogen-cooled isopentane. Small samples of gastrocnemius and soleus muscles were fixed for transmission electron microscopy (TEM) as previously described [49]. The post-mortem histopathological diagnosis of PDAC was made by two independent experienced investigators, and triple transgenic mice without PDAC were excluded from the study. In addition, WT mice were assessed for normal pancreatic histology. The study was approved by the Regional Commission Giessen (MR 20/11-Nr.70/2009) and performed in compliance with the regulations for animal experiments at the Philipps-University Marburg.

### 2.2. Histological Examination

For lectin-staining [53], cross-sections (8 μm) of snap-frozen gastrocnemius and soleus muscles were cut in a cryostat, fixed in 4% paraformaldehyde/phosphate-buffered saline (PFA/PBS) for 10 min at room temperature. Hydrogen peroxide (0.05% in PBS; pH 7.4) was used to block endogenous peroxidases. Then, cross-sections were incubated (30 min; 37 °C) with 40 μg/mL horseradish-peroxidase conjugated Isolectin B4 of Bandeiraea simplicifolia (BSI–B4) (Merck-Sigma-Aldrich Co. LLC, St. Louis, MO, USA) in PBS. A negative control with D-Galactose was performed. Thereafter, cross-sections were washed with PBS, incubated in a solution of 25 mg 3-3′-diaminobenzidine (Merck-Sigma), 50 µL hydrogen peroxide 30%, solved in 100 mL PBS, rinsed with PBS, and immediately counterstained with Mayer’s hematoxylin (Carl Roth GmbH, Karlsruhe, Germany).

**Figure 2:**
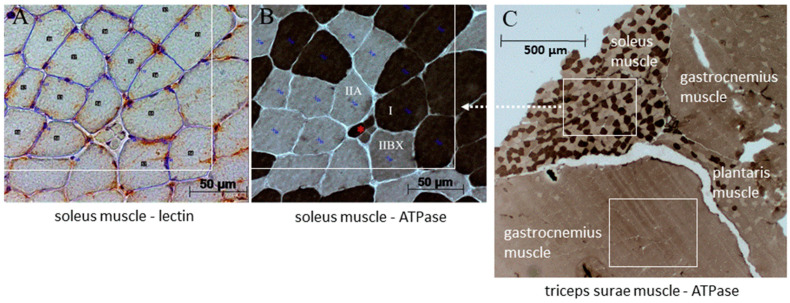
Examples of two serial cross-sections of soleus muscle showing lectin- (**A**) and corresponding ATPase-staining (**B**). Fibers in the lectin-staining are edged in blue and the brown capillaries are marked with crosses (**A**). The three distinguishable fiber types (I, IIA, IIBX) in the ATPase-staining are indicated in (**B**). * Symbol indicates a muscle spindle. Representative locations of the region of interest (edged by a rectangle) are indicated for the soleus and gastrocnemius muscle within the triceps surae muscle stained for ATPase (**C**).

Fiber types I, IIA, and IIBX were identified according to the acid-sensitive activity of their adenosine triphosphatase (ATPase) in unfixed serial cross-sections (8 μm), as previously described [54,55] (Figure 2). Microscopic images of the ATPase- and lectin-stained cross-sections were recorded using an Axio Imager M2 microscope (Carl Zeiss GmbH, Oberkochen, Germany) including the high-resolution imaging system Axio-Cam HRc/AxioVision Rel. 4.8 (Carl Zeiss GmbH). After ATPase-staining or lectin-staining, serial cross-sections with at least 100 fibers were selected and edged by a rectangle (region of interest—ROI) (Figure 2A,B). Only fibers with a cross-sectional area (CSA) >50% within the ROI were examined. Histomorphological parameters assessed were fiber-type-specific CSA, minimal Feret diameter (minFeret), fraction of total fiber population, capillary contacts, and centronucleation. For capillary density, only capillaries inside the rectangle were considered. For fiber-capillary contacts, all capillaries around the fibers were considered. Image analyses were performed using the ImageJ/Fiji (National Institute of Health, Bethesda, MD, USA a.o.). Analyses in the gastrocnemius muscle were limited to its large homogenous ‘white’ (i.e., superficial) area consisting of type IIBX only, since its soleus muscle-adjacent portion may variably contain type IIA, and sometimes type I fibers (Figure 2C).

For immunohistochemistry of MAO-A expression, gastrocnemius muscle cryo-cross-sections were fixed by 5% paraformaldehyde (10 min), blocked with bovine serum albumin (BSA), and incubated with MAO-A monoclonal rabbit IgG antibody (Abcam, Cambridge, UK; 1:100) detected by an anti-rabbit secondary antibody that was conjugated with horseradish peroxidase (HRP) for metabolization of 3,3′-diaminobenzidine (DAB) with H_2_O_2_ as chromogene substrate. Nuclei were counterstained with Mayer’s hematoxylin (Carl Roth GmbH, Germany). In digital images (200×) obtained by the Zeiss Axio Imager.M2 microscope (Carl Zeiss AG; Oberkochen, Germany) combined with Axio-Cam HRc/AxioVision (Carl Zeiss GmbH), the MAO-A positive area in terms of percentage of intact muscle tissue was quantified in two representative areas with >150 type IIBX fibers using ImageJ/Fiji.

To evaluate NMJ integrity (innervation status) in quadriceps muscle, every 6th out of 60 serial cryo-cross-sections (8 µm), i.e., 10 cross-sections per mouse were used for pre- and post-synaptic NMJ co-staining in subgroups (*n* = 5 WT, *n* = 4 PDAC, *n* = 5 WT with HH and *n* = 5 PDAC with HH). After fixation with 4% PFA/PBS and block by 1% BSA/PBS for 30 min, PBS-washed cross-sections were incubated in a humidified chamber overnight with biotin-XX-conjugated α-bungarotoxin (BTX, 1:500; Invitrogen, Eugene, OR 97402, USA) for post-synaptic staining, and an antibody against the vesicular acetylcholine transporter (vAChT antibody (1:1000, Lee Eiden, 80259, Lot No.: bl. 6/97) for pre-synaptic staining. After washing, cross-sections were incubated for 2 h with Cy3-conjugated streptavidin (1:200, Dianova GmbH, Hamburg, Germany) or Alexa Fluor^®^488 labeled donkey anti-rabbit IgG (1:200, MoBiTec GmbH, Göttingen, Germany) for the detection of BTX and vAChT antibodies, respectively. Slides were mounted using Immu-Mount™ (Fisher Scientific GmbH, Schwerte, Germany) and glass coverslips. Cross-sections were treated identically except for incubation with BTX or the primary vAChT antibodies served as controls. Con-focal images were collected with a C2 system on an Eclipse Ti2 inverted microscope (Ni-kon GmbH, Düsseldorf, Germany) using the software NIS-Elements AR 4.30.01 (Nikon GmbH). Each NMJ was scanned in a 630-fold magnification at 250 Hz with an image size of 1024 *×* 1024 pixels. Subsequently, Fiji software was used for morphometrical analysis, determining the BTX and vAChT immunolabeled areas, as well as their overlap area.

To assess the NMJ density of BTX+ post-synapses, every 7th cross-section was treated for 10 min with 3% H_2_O_2_ to block endogenous peroxidases, and thereafter, post-synaptic NMJ were detected by incubation with biotin-XX-conjugated α-BTX and horseradish peroxidase (HRP)-conjugated streptavidin (Jackson ImmunoResearch Laboratories. Inc., West Grove, PA, USA) using DAB as a chromogen substrate. Nuclei were counterstained with Mayer’s hematoxylin (Carl Roth GmbH, Germany). NMJ count per area was determined in digital images (200-fold magnification) obtained by the Zeiss Axio Imager.M2 microscope combined with Axio-Cam HRc/AxioVision.

### 2.3. Mitochondrial Ultrastructure

Using TEM, the ultrastructural integrity of gastrocnemius and soleus muscle mitochondria was studied at 25,000-fold magnification as previously described [56], in 5–8 mice per WT, Ca, WTHH, or CaHH group and >10 images per animal. Mitochondrial ultrastructure was categorized as ‘damaged’ in the case of >50% loss of the cristae, and/or >50% disruption of the outer membrane, or as ‘normal’ otherwise. The ‘damaged’ fractions (%) of each muscle were compared between the groups.

### 2.4. Western Blotting of MAO-A Expression

Gastrocnemius samples were lysed using RIPA (radioimmunoprecipitation assay) buffer pH 7.5 (Cell Signaling Technology Europa, Leiden, The Netherlands), containing the protease/phosphatase inhibitor cocktail (Cell Signaling Technology, Boston, MA, USA). The total protein concentrations were determined using the Pierce BCA assay (bicinchoninic acid) (Thermo Scientific, Rockford, IL, USA) according to the manufacturer’s instructions. Proteins were loaded on pre-cast polyacrylamide NuPAGE^®^ 4–12% Bis-Tris gels (Life Technologies GmbH, Darmstadt, Germany). After SDS-PAGE, proteins were transferred onto 0.45 µm nitrocellulose membranes [Millipore (Billerica, MA, USA)]. Before performing the immunoreactions, the membranes were stained for 1 min with Ponceau S solution (0.1% in 3% trichloroacetic acid) at RT, and then rinsed with distilled water to remove the background and documented by the Fusion-SL Advance™ imaging system (Peqlab) according to the instruction manual. Membranes were destained in blocking buffer (5% milk in tris-buffered saline [TBS]). Thereafter, membranes were incubated with the primary antibodies (rabbit monoclonal anti-Monoamine Oxidase A [MAO-A] antibody, ab126751, Abcam plc., Cambridge, UK) overnight at 4 °C in blocking buffer. After washing with TBS 0.1% Tween 20, membranes were incubated with ECL-anti-rabbit IgG-horseradish peroxidase (HRP) antibody (MA9340, GE Healthcare, Amersham, UK). Moreover, after washing, the peroxidase reaction was visualized with Immobilon^TM^ Western (HRP) Substrat (Merck Chemicals GmbH. Darmstadt, Germany). The intensity of WB bands and Ponceau S total protein was quantified using ImageJ/Fiji software from the National Institutes of Health (Bethesda, MD, USA). Total proteins according to Ponceau staining < 140 kDa were used to normalize the intensity of the bands [57].

### 2.5. Intramyocellular Glutathione (GSH) Content and Glutathione Redox State

Gastrocnemius muscle GSH content and glutathione redox state were determined according to Tietze (1969) [58] as previously described [59]. Briefly, 20–30 mg of muscle tissue were homogenized, deproteinized with 500 µL 2.5% sulfosalicylic acid (SSA), sonicated, and centrifugated for 10 min at 13,000× *g* and 5 °C. The supernatant was used to determine its content of total GSH (tGSH) and its oxidized disulfide GSSG form. Reduced GSH (rGSH) was calculated by subtraction of GSSG from tGSH. The total protein content of the pellet, corresponding to the supernatant volume was quantified by the colorimetric Bio-Rad protein assay (BioradLaboratories, Munich, Germany) to normalize the intracellular tGSH and GSSG.

### 2.6. Quantitative Reverse Transcription Polymerase Chain Reaction (qRT-PCR)

RNA was extracted from tissue using peqGOLDTriFast™ (VWR International GmbH, Darmstadt, Germany) according to the manufacturer’s instructions. RNA concentration was assessed using a NanoDrop 2000c spectrophotometer (Thermo Fisher Scientific Inc., Waltham, MA, USA) by optical density (OD) 260 nm, and purity by OD260 nm/OD280 nm. A ratio of 1.8 to 2.0 was accepted as pure. RNA integrity (RIN) was confirmed using a RNA 6000 NanoChip kit on an Agilent 2100 Bioanalyzer (Agilent Technologies Inc., Santa Clara, CA, USA). RNA samples with a RIN between 8 and 10 were considered as suitable for reverse transcription (RT). An aliquot of total RNA was treated with 1 unit DNAse (Thermo Fisher Scientific Inc.) (30 min; 37 °C). The treated RNA was employed to perform the RT for 1 h at 42 °C, using oligo primer (dT)_12–18_ (Agilent Technologies Inc.), 20 units of the reverse transcriptase, included in the Affinity Script multiple temperature cDNA synthesis kit (Agilent Technologies Inc.), 24 units of Ribo Lock™ RNAse inhibitor (Thermo Fisher Scientific Inc.), and 4 mM dNTP mix (Agilent Technologies Inc.). The cDNA was used for RT-qPCR using the QuantiTect-primer assays (Table 1) (Qiagen N.V., Venlo, The Netherlands) along with Takyon™ Low Rox Probe Master-Mix dTTP Blue (Eurogentec, Seraing, Belgium) or Agilent™ Brilliant III Ultra-Fast SYBR^®^ Green QPCR Master-Mix (Agilent Technologies Inc.). The thermal profile consisted of 3 min at 95 °C followed by 45 cycles at 95 °C for 10 s and 60 °C for 20 s. The qPCR and data analyses were performed using the Stratagene Mx3005P™ qPCR System (Agilent Technologies Inc.). For each unknown sample, the relative amount was calculated by linear regression analysis from their respective standard curves, which were generated from a pool of cDNA. Specificity of the amplified product was confirmed by the melting curve analysis (55–95 °C). The expression of housekeeping (Table 1) genes (RER1 for soleus muscle and β-actin for gastrocnemius muscle) were selected with the use of the software Normfinder [60].

### 2.7. Statistical Analyses

Statistical analyses were performed using R Deducer [https://www.R-project.org/]. Shapiro-Wilk normality test, *Levene’s* test for homogeneity of *variance*, two-factorial ANOVA, and Tukey’s test were performed. Two-factorial ANOVA was applied to the total study population to assess the effect of PDAC (Ca and CaHH group) vs. WT (WT and WTHH groups) as well as the effect of HH (WTHH and CaHH group) vs. untreated mice (WT and Ca group) with significant differences being presented in separate boxes within the graphs. Significant differences between two groups (post hoc) are indicated within the graph by x (for PDAC effects) or # (for HH effects). A significance level of ≤5% was chosen for this study.

## 3. Results

The average weight of the mice was between 24.03 and 27.88 g, with no significant difference between groups. Concerning age, a significant difference by PDAC and an interaction existed by ANOVA. In the post-hoc test, all groups were significantly different in age, except for the comparison between CaHH and WTHH (Ca—WT *p* < 0.001; WTHH—WT *p* < 0.01; CaHH—WT *p* < 0.05; WTHH—Ca *p* < 0.01; CaHH—Ca *p* < 0.01) (Table 2).

### 3.1. Gastrocnemius Muscle

**Figure 3:**
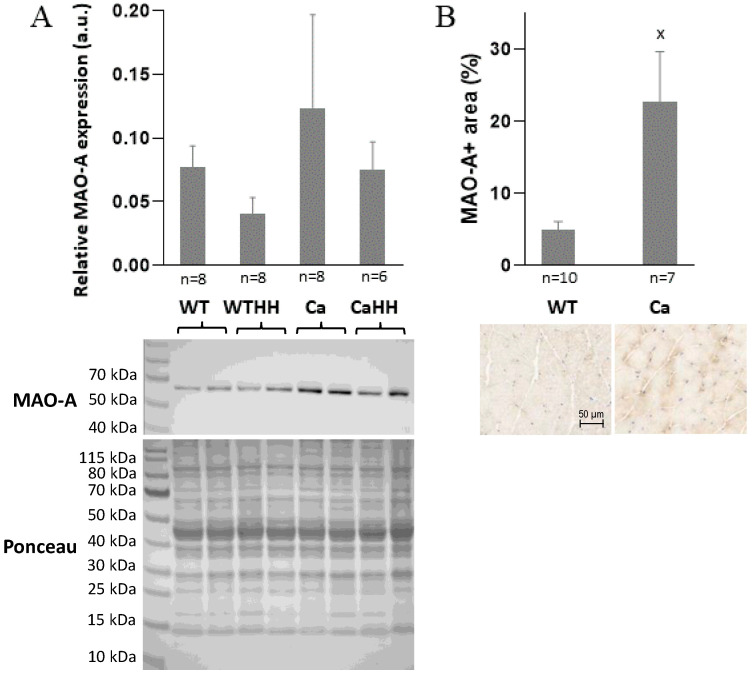
(**A**) Relative MAO-A expression in gastrocnemius muscle in WT and PDAC-bearing (Ca) mice with or without HH treatment as determined by Western blot with normalization for total protein abundance by Ponceau staining (see Section 2). (**B**) Percentage of MAO-A positive area by immunohistochemistry of gastrocnemius cryo-cross-sections in the WT and the Ca group with representative examples below. Data are mean ± SEM, ^x^ *p* < 0.05.

As a target for HH treatment, MAO-A expression was confirmed by Western blot with normalization for total protein in gastrocnemius muscle of all four groups, i.e., WT, Ca WTHH, and CaHH (Figure 3A). There was a trend toward a PDAC-related increase in the highly variable MAO-A expression, which tended to decrease with HH treatment in both WT and PDAC-bearing mice. In addition, immunohistochemistry revealed a significantly higher MAO-A expression in Ca compared to WT mice (Figure 3B). Notably, no data were obtained regarding MAO-A enzyme activity and its change with HH treatment.

**Figure 4:**
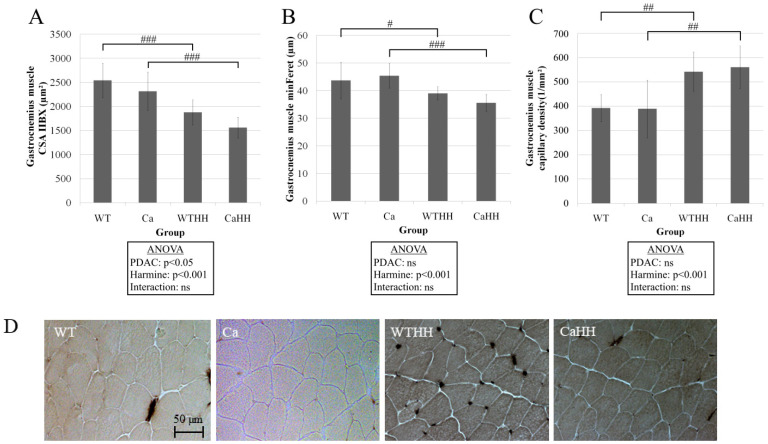
(**A**) CSA of fiber type IIBX of the gastrocnemius muscle. ANOVA revealed a significant reduction in CSA triggered by PDAC (Ca) or HH treatment. (**B**) Corresponding minFeret of the fiber type IIBX of the gastrocnemius muscle. A significant reduction by the HH treatment can be seen. In contrast to the CSA, no PDAC effect was found. (**C**) Reduced CSA led to an increased capillary density triggered by the HH effect (^#^ *p* < 0.05; ^##^ *p* < 0.01; ^###^ *p* < 0.001; ns, not significant; wild type mice (WT), *n* = 9; PDAC mice (Ca), *n* = 7; WT mice with HH treatment (WTHH), *n* = 11; PDAC mice with HH treatment (CaHH), *n* = 6). Mean values ± SD and ANOVA results are shown. (**D**) Representative examples of ATPase-staining for male WT, Ca, WTHH, and CaHH mice in gastrocnemius muscle regions solely containing type IIBX fibers.

PDAC (*p* < 0.05) and HH (*p* < 0.001) led to a significant decrease in type IIBX CSA (Figure 4). A significant HH-mediated, as well as a PDAC-induced, reduction in CSA was found (Figure 4A). Additionally, HH treatment led to a decrease (Ca—CaHH: −9.85 µm; WT—WTHH: −4.64 µm) in the minFeret (Figure 4B), which is a CSA-corresponding length indicating the shortest distance between two parallel tangents on the fiber diameter. Thereby, in line with fiber atrophy, HH treatment significantly increased capillary density by 38.10% in WT and by 44.21% in PDAC mice (Figure 4C), while capillary fiber contacts remained unchanged (not shown).

Moreover, RT-qPCR analyses revealed that neither the expression of apoptosis-relevant genes (BAX, Caspase 3, BCL-2) nor E3 ligases MuRF1 and FBXO32, or of MAO-A and MAO-B were significantly altered with PDAC or HH. Similarly, the expression of proinflammatory genes, such as TNFα, IL-6, and COX2 showed no related differences (Table 3).

**Figure 5:**
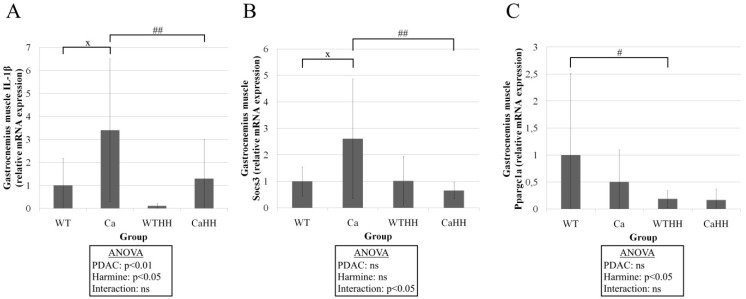
(**A**) Relative mRNA expression of IL-1β in the gastrocnemius muscle with a PDAC triggered increase in Ca compared to WT mice, and HH triggered decrease in CaHH compared to Ca mice. (**B**) Relative mRNA expression of Socs3 showed an interaction between PDAC and HH with an increase in mRNA expression in Ca compared to WT mice, and a decrease in mRNA expression in CaHH compared to Ca mice. (**C**) Relative mRNA expression of Ppargc1a showed the HH effect with a decrease in mRNA expression in WTHH compared to WT mice. PDAC effect (^x^ *p* < 0.05); HH effect (^#^ *p* < 0.05; ^##^ *p* < 0.01); ns, not significant. WT, *n* = 9; Ca, *n* = 11; WTHH, *n* = 11; CaHH, *n* = 7. Mean values ± SD and ANOVA results are shown.

However, IL-1β expression was found to be significantly increased by PDAC (*p* < 0.01) and, importantly, significantly decreased by HH (*p* < 0.05) (Figure 5A).

Furthermore, ANOVA revealed a non-synergistic interaction at the mRNA expression of Socs3, increasing in Ca compared to WT mice by 160.43% and decreasing in CaHH compared to Ca mice by 195.7% (Figure 5B). As another significant effect in gastrocnemius muscle, HH led to a decreased mRNA expression of Ppargc1a by 81.52% in WTHH compared to WT mice (Figure 5C).

Analyses of GSH content and redox state in gastrocnemius muscle homogenate by ANOVA revealed (Table 4) that, overall, PDAC had no significant impact. However, HH treatment led to a significant decrease in total (WT—WTHH *p* < 0.001; WT—CaHH *p* < 0.05) as well as in reduced GSH (WT—WTHH *p* < 0.05). Since HH and PDAC tended to decrease GSSG as well (WT—Ca *p* < 0.01; WT—WTHH *p* < 0.001), the ratio of reduced GSH to GSSG (WT—Ca *p* < 0.05; Ca—WTHH *p* < 0.05) was not significantly changed by HH. Overall, HH appeared to impair intra(myo)cellular GSH availability, however, without a major shift in oxidation status.

### 3.2. Soleus Muscle—Histomorphometry and Gene Expression

**Figure 6:**
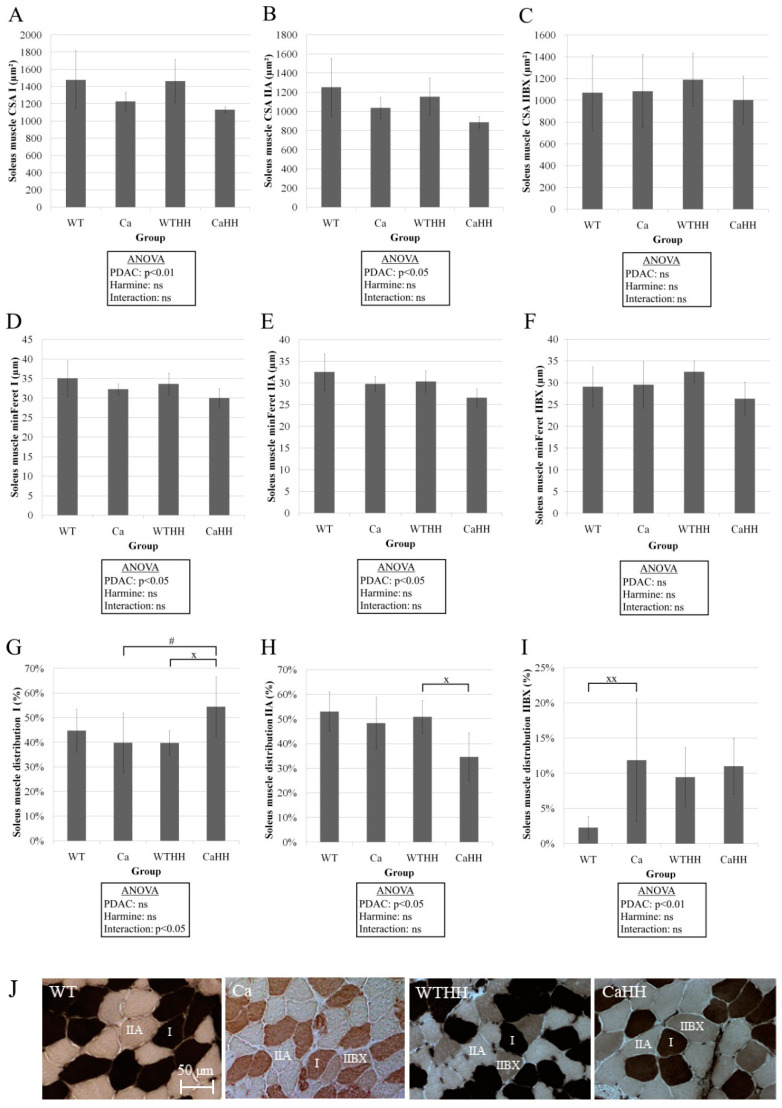
(**A**–**C**) CSA of the fiber types I, IIA, and IIBX of the soleus muscle. In types I and IIA, a reduction in CSA by the PDAC could be found using ANOVA. CSA of the type IIBX fibers was not altered. (**D**–**F**) Corresponding minFeret of the fiber types I, IIA, and IIBX of the soleus muscle. Similar to CSA, a shortage in types I and IIA was found using ANOVA. (**G**–**I**) Distribution of the three fiber types of the soleus muscle. PDAC and a PDAC–HH interaction caused a fiber shift from type IIA to types I and IIBX. PDAC effect (^x^ *p* < 0.05; ^xx^ *p* < 0.01); HH effect (# *p* < 0.05); ns, not significant. *n* = 29, WT = 11, Ca = 8, WTHH = 6, CaHH = 4. Mean values ± SD and ANOVA results are shown. (**J**) Representative examples for soleus muscle fiber typing by ATPase-staining in WT, Ca, WTHH, and CaHH mice.

In soleus muscle, PDAC led to a decrease in CSA in fiber types I (*p* < 0.01) and IIA (*p* < 0.05) (Figure 6A,B) and to a corresponding reduction in minFeret in fiber types I (*p* < 0.05) and IIA (*p* < 0.05) (Figure 6D,E). Furthermore, interaction of PDAC and HH resulted in a significantly (*p* < 0.05) increased number of type I fibers by 14.59% in soleus muscle of CaHH mice in comparison to the WTHH or Ca group (Figure 6G). Driven by PDAC, the number of type IIA fibers in soleus muscle of CaHH mice significantly (*p* < 0.05) decreased by 16.27% compared to the WTHH group (Figure 6H). In soleus muscle of PDAC mice, the number of type IIBX fibers significantly (*p* < 0.01) increased by 9.57% compared to WT mice (Figure 6I). Capillary density and fiber-capillary contacts were similar in all groups under test (Table 5).

**Figure 7:**
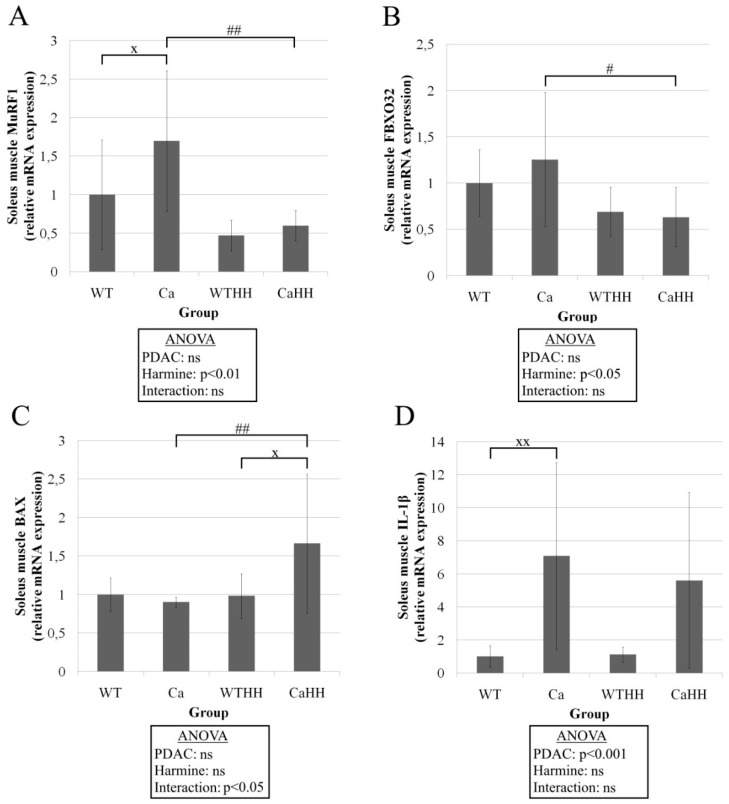
Gene expressions in soleus muscle. (**A**) MuRF1, (**B**) FBXO32, (**C**) BAX, (**D**) IL-1β; WT, *n* = 11; Ca, *n* = 7; WTHH, *n* = 6; CaHH, *n* = 6; PDAC effect (^x^ *p* < 0.05; ^xx^ *p* < 0.01); HH effect (^#^ *p* < 0.05; ^##^ *p* < 0.01); ns, not significant. Mean values ± SD and ANOVA results are shown.

Regarding relevant proteolytic signals and markers in soleus muscle, a PDAC-driven increase in MuRF1 mRNA, but not in FBXO32 mRNA expression was found (Figure 7A). As a potentially anti-cachectic effect, HH treatment led to a significant decrease in MuRF1 and FBXO32 expression by 64.78% (*p* < 0.01) and 49.55% (*p* < 0.05), respectively (Figure 7B). Due to an interaction between PDAC and HH (*p* < 0.05), BAX expression in CaHH significantly increased by 68.81% (*p* < 0.05) compared to WTHH mice and by 84.07% (*p* < 0.01) compared to Ca mice (Figure 7C). ANOVA and Tukey’s test showed a significant (*p* < 0.001) increase in IL-1β expression driven by PDAC (Figure 7D). As with gastrocnemius muscle, Caspase 3, BCL-2, MAO-A, and MAO-B gene expressions were unchanged in soleus muscle (Table 6). The expressions of proinflammatory CD68, TNFα, IL-6, and COX2 genes were not altered, as well (Table 6). However, HH led to a significant downregulation of MMP9 overall. Furthermore, MyoG was found to be significantly upregulated through HH by interaction with PDAC.

### 3.3. Gastrocnemius and Soleus Muscles—Mitochondrial Integrity and Centronucleation

In gastrocnemius muscle, no significant global effect of PDAC (*p* = 0.595) or HH (*p* = 0.160) was detected regarding the mitochondrial integrity as assessed by TEM. Among the total mitochondrial number analyzed per animal in WT (*n* = 220.0 ± 50.4), Ca (*n* = 230.8 ± 44.9), WTHH (*n* = 137.5 ± 13.0), and CaHH (*n* = 153.1 ± 14.8), the ‘damaged’ fractions amounted to 39.8% ± 3.7%, 37.8% ± 4.3%, 42.5% ± 5.8%, and 50.1% ± 5.0%, respectively.

Similarly, no significant global effect of PDAC (*p* = 0.513) or HH (*p* = 0.075) was found regarding the mitochondrial integrity in soleus muscle. Among the total mitochondrial number analyzed per animal in WT (*n* = 364.6 ± 25.7), Ca (*n* = 311.3 ± 42.0), WTHH (*n* = 307.5 ± 49.3), and CaHH (*n* = 370.3 ± 57.0), the ‘damaged’ fractions were 41.1% ± 3.1%, 39.4% ± 2.4%, 49.2% ± 4.4%, and 45.8% ± 4.40%, respectively.

However, a combined analysis of both muscles revealed a significant effect of HH (*p* = 0.022) but not PDAC (*p* = 0.930) in terms of increased mitochondrial damage.

No significant global effect of PDAC or HH was detected on the fraction of centronucleated cells in gastrocnemius muscle or within the total fiber population, type I or type IIA fibers in soleus muscle (Table 7). However, within type IIBX fibers of soleus muscle alone or in combination with gastrocnemius and soleus muscles, a significant increase in centronucleated fraction with HH but not PDAC was detected.

### 3.4. Quadriceps Muscle: Morphological NMJ Integrity

**Figure 8:**
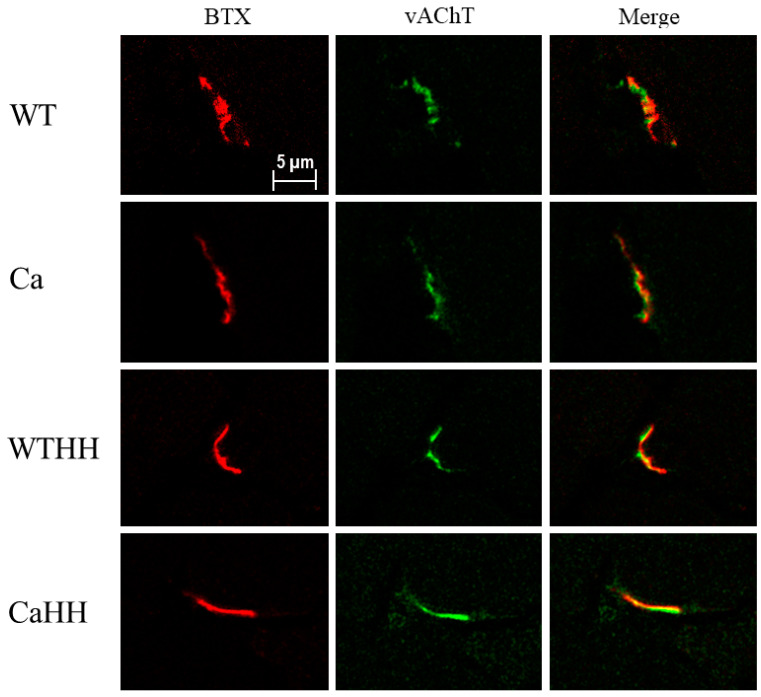
Representative examples of NMJ in quadriceps muscle for the WT, Ca, WTHH, and CaHH group, co-stained post-synapsis (BTX = α-Bungarotoxin) and pre-synapsis (vAChT = vesicular acetylcholine transporter) and their respective overlay.

Evaluation of NMJ integrity by pre-/post-synaptic co-staining in subgroups (Table 8, Figure 8) indicated that the post-synaptic BTX+ area was significantly reduced with HH treatment, while PDAC had no impact (Ca—CaHH *p* < 0.05). Moreover, there was a significant interaction of HH treatment with PDAC in a way that the HH treatment decreased vAChT+ area (Ca—CaHH *p* < 0.05) and the absolute percentage overlap of vAChT+ with BTX+ areas (Tukey’s test: vAchT/BTX overlap [µm^2^] Ca—CaHH *p* < 0.05; vACht/BTX overlap [%] WT—Ca *p* < 0.05). Furthermore, significant correlations were found between rGSH/GSSG ratios in gastrocnemius muscle and NMJ vAChT/BTX overlap [%] in quadriceps muscle (r = 0.580, *p* = 0.015), as well as between rGSH content and NMJ BTX+ area (r = 0.512 *p* = 0.035) of these muscles, which both consist of type IIBX fibers.

## 4. Discussion

In the presently studied triple transgenic KPC mouse model, a recently reported cachexia-inducing effect of PDAC was confirmed in terms of reduced CSA of muscle fiber type IIBX in gastrocnemius and, as a global effect across all groups, of fiber types I and IIA in soleus muscle [49]. No significant pro-atrophic impact of PDAC was, however, detected in soleus muscle IIBX fibers, which represented a rather small fraction only. Our data on PDAC-related muscle wasting in these most frequently studied muscle types [4,18,31,49] are in line with the pro-cachectic effect of various other carcinoma in [19,26,27,28,29,61], which may vary widely between different types of cancer, fiber composition, and other factors [18]. Notably, changes in body weight were detected neither with PDAC nor with HH treatment. The fact that it did not reflect significant muscle wasting induced by PDAC and, surprisingly, also with HH in gastrocnemius muscle, may be due to concomitant variable changes in fat mass and increases in ascites fluid volume and/or tumor mass, all of which could not be measured.

Regarding fiber type distribution, a shift toward types I and IIBX in soleus muscle was observed solely due to a combined PDAC and HH effect. Alterations in fiber type composition with cancer cachexia may involve apoptosis, fiber transition, or impaired fiber type regeneration [18], all of which may contribute to differences and contradiction between findings on fiber composition with cancer [19,25,26,27,28,29]. It is very likely that the significant increase in capillary density, as presently observed, results from the reduction in fiber CSA in the gastrocnemius muscle, as changes in capillary-fiber contacts were absent.

Muscle IL-1β, a predictor of cachexia and higher mortality rates in patients with PDAC [62], was the only consistent parameter that was increased in both muscles under study and might have triggered acute phase reaction leading to cachexia [63]. In gastrocnemius muscle, a concomitant upregulation of Socs3 was found, that might be linked to the higher IL-1β [64,65], whereas in soleus muscle, MuRF1 was found to be increased as a protein-catabolic signal. No significant PDAC-related changes were detected in the expression of Ppargc1a, the role of which in metabolism and prognosis of cancer cachexia is still controversial [66,67,68,69].

As a presently addressed target of HH, skeletal muscle MAO-A was shown by Western blot (gastrocnemius muscle) and RT-qPCR (soleus and gastrocnemius muscles) to be expressed in both WT and PDAC mice. Thereby, a PDAC-related increase in MAO-A expression was neither detected on the RNA level nor on the protein level. However, there was a trend toward higher MAO-A protein expression with PDAC in Western blot and immunochemistry revealed significantly higher MAO-A expression in Ca compared to WT. These data reveal high variability and are only partly in line with a previous study showing a massive MAO-A upregulation along with increased H_2_O_2_ production upon glucocorticoid treatment in myocytes in vitro [32]. Even in the absence of significant changes in MAO-A expression, the present HH treatment can, however, be expected to substantially inhibit the enzyme activity of MAO-A, thereby decreasing H_2_O_2_ production as a pro-inflammatory and -cachectic signal within skeletal muscle.

Surprisingly, however, rather than reversing muscle atrophy, HH treatment led to a (further) reduced CSA of type IIBX fibers in the gastrocnemius muscle. As a serious adverse HH effect in the context of cachexia that is not described before, it occurred despite the observed significant reduction in the likely pro-cachectic IL-1β mRNA expression in gastrocnemius muscle. This protective anti-inflammatory effect may be in line with previous findings in human neurons after death following traumatic brain injury [70], or in mice serum [71,72]. Importantly, HH treatment also decreased the expression of MuRF1 and FBXO32 in soleus muscle, but was not able to diminish the PDAC-triggered muscle atrophy. Therefore, this, in theory, anti-cachectic, i.e., beneficial HH effect presently appeared to be overridden by another consequence of HH treatment. It cannot be excluded that apoptosis as suggested by BAX upregulation with HH (by interaction with PDAC) may have contributed to losses in muscle mass, as histomorphometry assesses fiber atrophy only.

Though obtained in subgroups only, our present data on NMJ integrity in the quadriceps muscle as another IIBX fiber-containing muscle suggest that NMJ may be compromised through HH, especially in PDAC mice. Of interest, IL-1β and possibly increased MAO-A activity is required for regeneration after peripheral nerve injury [73,74], while inhibition of MAO-B, not achieved by HH, may be beneficial for regeneration [75]. Therefore, anti-inflammatory effects through the presently applied HH dose may not be generally desirable in the context of cachexia treatment, though inhibition of MAO-A/B may reduce ROS production following denervation [76]. It remains to be studied as to what extent the adverse effect of HH is dose-dependent and overrides a beneficial anti-cachectic effect through downregulation of E3-ligases and IL-1β.

The assumption of compromised innervation through HH is presently supported by the finding that tGSH and rGSH availability in gastrocnemius muscle decreased significantly with HH treatment [73,76,77] and, furthermore, by the finding of increased centronucleation [78] in soleus type IIBX, or combined gastrocnemius and soleus IIBX fibers. In addition, as a source of ROS, dysfunctional mitochondria have indeed been implicated as a cause or a consequence of muscle denervation [76,79]. Increased ROS production of dysfunctional mitochondria may not necessarily be associated with detectable ultrastructural damage [18,80]. However, we presently detected an overall increase in the fraction of damaged mitochondria through HH when considering the combined data of both muscles under study, while PDAC was without any significant effect. Whether the adverse effect of HH primarily targets the NMJ or mitochondria remains open at present, however, both may lead to oxidative shifts in intracellular GSH status and proteasomal activation as a major cause of fiber atrophy. Notably, in the context of its possible adverse effects, cytotoxic effects of HH have also been described in pulmonal fibroblasts [81] and hepatocytes [82]. Possible triggers may be the ability of many β-carbolines to intercalate with DNA [83,84] and to provoke a G2 phase arrest [85,86] by the inhibition of topoisomerase I [87] or cyclin-dependent kinase [88]. More generally, it is noteworthy that HH may induce psychomotor alterations, such as reduced physical activity and anhedonia in rats, which presently cannot be excluded as a possible explanation for the HH-related muscle atrophy [89].

Importantly, the presently reported effects through HH may also, at least in part, be conveyed via HH-related inhibition of the x-chromosomal multifunctional dual-specificity tyrosine phosphorylation regulated kinases 1A (DYRK1A) and the 1B (DYRK1B/MIRK). DYRK1A is required for normal brain development and axonal transport and is expressed in adult NMJ [90], where DYRK1A inhibitors, such as HH might possibly have adverse effects, though data in this respect are lacking. However, since DYRK1A overexpression in Down’s Syndrome (DS) is strongly implicated in DS-associated oxidative stress, Alzheimer’s disease [91], and motor dysfunction [90], DYRK1A inhibitors may be beneficial in this condition, e.g., by upregulating the nuclear factor erythroid 2-like 2 (NRF2) [92]. However, DYRK1B is mainly expressed in skeletal muscle, where it plays a role in antioxidative defense [93,94] somewhat in contrast to the role of MAO-A. Therefore, HH may differentially alter the oxidative milieu through MAO-A and DYRK1B inhibition. However, since myocellular DYRK1B expression is critical for myogenesis, limited apoptosis, myocellular fusion, fiber differentiation, antioxidative defense, and autophagy (e.g., of damaged mitochondria) [94,95], its inhibition by HH may contribute to the presently observed muscle alterations, such as centronucleation, mitochondrial damage, fiber shift, and atrophy. Given that MAO-A was presently found to be upregulated in quadriceps muscle, the HH-related reversal of pro-cachectic PDAC effects (i.e., lowering IL-1β in gastrocnemius and MURF1 in soleus muscle) may be attributable to MOA-O inhibition, i.e., to reducing oxidative stress rather than to its augmentation via DYRK1B (DYRK1A) inhibition. Certainly, further studies on the relative contribution of these players to cachexia development and to therapeutic benefit or adverse events of HH are warranted.

Owing to the limited sample size, some gender imbalance between the groups, and the lack of a sham control, the present study may overall be considered as hypothesis-generating, especially with regard to soleus muscle histomorphometry in both HH-treated groups and the small subgroups explored for mitochondrial and NMJ integrity. Due to limitations in tissue/blood samples for further Western/ELISA analyses, the present results were mainly based on muscle qRT-PCR and histomorphometry, and thus unable to assess a potential contribution of systemic inflammation to the effect of PDAC or HH.

## 5. Conclusions

This hypothesis-generating study provides first insight into an understudied topic, i.e., MAO-A expression with cancer-related muscle wasting and the therapeutic potential of the MAO-A inhibition with HH. Using a transgenic mouse model of PDAC with confirmed fiber atrophy, we show a desirable inhibiting effect of HH on inflammation (IL-1β and Socs3) in gastrocnemius muscle and on signals of fiber atrophy via proteolysis (MuRF1 and FBX032), however, with a possible pro-apoptotic effect (BAX). Overall, this, however, did not translate into inhibition of PDAC-related muscle fiber atrophy. Rather, HH promotes fiber atrophy, at least in ‘white’ glycolytic muscle. Our preliminary data indicate that this adverse effect of HH may arise from compromised innervations, as also supported by GSH depletion, mitochondrial damage, and centronucleation. Notably, these adverse prooxidative effects impairing neuromuscular maintenance may also arise from HH-related inhibition of neuronal DYRK1A and/or DYRK1B. Whether different HH dosages may more effectively reduce inflammatory and fiber atrophy signals while avoiding neuromuscular adverse effects warrants further studies. In this context, it would be interesting to investigate the impact of other commonly used MAO inhibitors, such as Moclobemide, Rasagiline, or Selegiline in the present experimental animal model.

## Figures and Tables

**Table 1:** QuantiTect primer assays used for qPCR.

Primer Assay	Symbol	Amplicon Length (bp)	Cat. No.
Actin Beta	ACTB	77	QT01136772
BCL2 Associated X, Apoptosis Regulator	BAX	76	QT00102536
BCL2 Apoptosis Regulator	BCL-2	104	QT02392292
Caspase 3	Caspase3	150	QT01164779
CD68 Molecule	CD68	67	QT00254051
F-Box Protein 32	FBXO32	103	QT00158543
Interleukin 1 Beta	IL-1β	150	QT01048355
Interleukin 6	IL-6	128	QT00098875
Monoamine Oxidase A	MAO-A	81	QT00109326
Monoamine Oxidase B	MAO-B	93	QT00145124
Matrix Metallopeptidase 9	MMP9	84	QT00108815
Myogenin	Myog	115	QT00112378
Paired Box Protein 7	PAX7	135	QT00147728
PPARG Coactivator 1 Alpha	Ppargc1a	63	QT00156303
Prostaglandin-Endoperoxide Synthase 2	PTGS2 (COX2)	95	QT00165347
Retention of Endoplasmic Reticulum protein 1 (S. cerevisiae)	RER1	86	QT00146580
Suppressor of Cytokine Signaling 3	SOCS3	90	QT00156303
Sequestosome 1	Sqstm1 (P62)	91	QT00127855
TATA Box Binding Protein	TBP	114	QT00198443
Tumor Necrosis Factor	TNFα	112	QT00104006
Tripartite Motif Containing 63	TRIM63(MuRF1)	116	QT00291991
Vascular Endothelial Growth Factor A	VEGFA	117	QT00160769

All Primers were purchased at Qiagen N.V., Venlo, The Netherlands.

**Table 2:** Group characteristics of untreated and treated WT and PDAC mice.

	WT	Ca	WTHH	CaHH	ANOVA		
					PDAC	HH	Interaction
n (m/f)	11(5/6)	11 (7/4)	11 (8/3)	7 (4/3)	-	-	-
weight (g)	27.88 ± 6.16	27.65 ± 3.47	27.17 ± 4.52	24.03 ± 3.16	*p* = 0.314	*p* = 0.186	*p* = 0.332
age(months)	3.91 ± 0.54	5.42 ± 0.77	4.61 ± 0.11	4.50 ± 0.36	*p* < 0.001	*p* = 0.552	*p* < 0.001

Shown are body weight, age, and total number (n) of male (m) and female (f) wild type (WT) or pancreatic ductal adenocarcinoma (Ca) mice with/without HH. Mean values ± SD and ANOVA results are shown.

**Table 3:** Additional RT-qPCR data of the gastrocnemius muscle.

		WT	Ca	WTHH	CaHH		ANOVA	
						PDAC	HH	Interaction
	n (m/f)	9 (4/5)	11 (7/4)	11 (8/3)	7 (4/3)	-	-	-
Apoptosis/Atrophy	BAX	1 ± 1.57	0.65 ± 0.67	0.42 ± 0.58	0.73 ± 0.69	*p* = 0.902	*p* = 0.406	*p* = 0.327
	BCL-2	1 ± 0.57	1.06 ± 0.58	1.62 ± 2.19	1.11 ± 0.85	*p* = 0.662	*p* = 0.441	*p* = 0.535
	Caspase 3	1 ± 1.09	0.79 ± 0.44	0.69 ± 0.36	0.92 ± 0.82	*p* = 0.977	*p* = 0.661	*p* = 0.388
	MuRF1	1 ± 2.45	0.14 ± 0.16	0.19 ± 0.53	0.11 ± 0.11	*p* = 0.252	*p* = 0.298	*p* = 0.371
	Fbxo32	1 ± 0.82	2.36 ± 2.84	0.57 ± 0.31	0.87 ± 0.43	*p* = 0.128	*p* = 0.107	*p* = 0.349
	MAO-A	1 ± 1.44	0.55 ± 0.49	0.20 ± 0.10	0.52 ± 0.54	*p* = 0.743	*p* = 0.113	*p* = 0.168
	MAO-B	1 ± 1.52	0.45 ± 0.54	0.21 ± 0.31	0.33 ± 0.31	*p* = 0.407	*p* = 0.104	*p* = 0.253
	MMP9	1 ± 2.43	0.25 ± 0.23	0.17 ± 0.37	0.15 ± 0.18	*p* = 0.337	*p* = 0.252	*p* = 0.386
Inflammation	CD 68	1 ± 2.01	0.68 ± 0.69	0.64 ± 1.53	1.99 ± 2.63	*p* = 0.457	*p* = 0.499	*p* = 0.180
	TNFα	1 ± 0.99	1.65 ± 2.15	0.53 ± 0.51	0.51 ± 0.45	*p* = 0.461	*p* = 0.093	*p* = 0.466
	IL-6	1 ± 1.52	0.83 ± 0.77	0.30 ± 0.58	1.24 ± 2.29	*p* = 0.461	*p* = 0.689	*p* = 0.239
	COX2	1 ± 1.06	1.45 ± 2.27	0.60 ± 0.62	0.20 ± 0.36	*p* = 0.903	*p* = 0.105	*p* = 0.381

Total number (n) of male (m) and female (f) wild type (WT) or pancreatic ductal adenocarcinoma (Ca) mice with/without HH treatment. Mean values ± SD and ANOVA results are shown.

**Table 4:** Intracellular glutathione content and redox state in gastrocnemius muscle.

	WT	Ca	WTHH	CaHH	ANOVA		
					PDAC	HH	Interaction
n (m/f)	11 (6/5)	11 (7/4)	10 (7/3)	4 (2/2)	-	-	-
tGSH (pmol/g)	10.40 ± 0.41	8.41 ± 0.81	5.32 ± 0.98	5.45 ± 1.97	*p* = 0.342	*p* < 0.001	*p* = 0.281
rGSH (pmol/g)	7.89 ± 0.44	7.07 ± 0.83	4.03 ± 0.94	3.66 ± 1.81	*p* = 0.536	*p* = 0.001	*p* = 0.811
GSSG (pmol/g)	2.51 ± 0.18	1.35 ± 0.19	1.29 ± 0.14	1.79 ± 0.69	*p* = 0.189	*p* = 0.129	*p* = 0.002
rGSH/GSSG (ratio)	3.31 ± 0.34	6.25 ± 0.92	3.18 ± 0.84	3.88 ± 1.94	*p* = 0.062	*p* = 0.192	*p* = 0.241

Total number (n) of male (m) and female (f) wild type (WT) or pancreatic ductal adenocarcinoma (Ca) mice with/without HH treatment. Data given as mean values ± SD, GSH = glutathione; GSSG = glutathione disulfide. *p*-values by two-factorial ANOVA applied to the total study population are given on the right.

**Table 5:** Additional histomorphometric parameters of the soleus muscle.

	WT	Ca	WTHH	CaHH	ANOVA		
					PDAC	HH	Interaction
n (m/f)	11 (5/6)	8 (5/3)	6 (6/0)	4 (2/2)	-	-	-
CSA (µm^2^)	1351 ± 299	1154 ± 86	1286 ± 199	1037 ± 31	*p* = 0.019	*p* = 0.338	*p* = 0.777
minFeret (µm)	33.66 ± 4.11	31.15 ± 1.48	31.75 ± 2.33	28.54 ± 2.33	*p* = 0.032	*p* = 0.092	*p* = 0.789
capillary contacts/all fiber types (n/fiber)	4.35 ± 0.81	4.49 ± 0.73	4.39 ± 0.49	4.02 ± 0.38	*p* = 0.896	*p* = 0.573	*p* = 0.383
capillary contacts/fiber type I (n/fiber)	4.50 ± 0.90	4.57 ± 0.81	4.56 ± 0.54	4.17 ± 0.45	*p* = 0.791	*p* = 0.683	*p* = 0.483
capillary contacts/fiber type IIA (n/fiber)	4.23 ± 0.79	4.37 ± 0.67	4.25 ± 0.52	3.83 ± 0.27	*p* = 0.861	*p* = 0.449	*p* = 0.323
capillary contacts/fiber type IIBX (n/fiber)	4.08 ± 1.07	4.26 ± 0.98	4.47 ± 0.62	3.70 ± 0.55	*p* = 0.705	*p* = 0.999	*p* = 0.233
capillary density (n/mm^2^)	1144 ± 176	1265 ± 246	1186 ± 308	1248 ± 141	*p* = 0.282	*p* = 0.851	*p* = 0.762

Total number (n) of male (m) and female (f) wild type (WT) or pancreatic ductal adenocarcinoma (Ca) mice with/without HH treatment. Fiber cross-sectional area (CSA). Mean values ± SD and ANOVA results are shown.

**Table 6:** RT-qPCR of the soleus muscle.

		WT	Ca	WTHH	CaHH	ANOVA		
						PDAC	HH	Interaction
	n (m/f)	11 (6/5)	7 (5/2)	6 (4/2)	6 (3/3)	-	-	-
Apoptosis/Atrophy	BCL-2	1 ± 0.52	0.94 ± 0.53	0.50 ± 0.36	0.70 ± 0.71	*p* = 0.836	*p* = 0.088	*p* = 0.564
	Caspase 3	1 ± 0.47	0.92 ± 0.37	1.22 ± 0.58	0.49 ± 0.42	*p* = 0.073	*p* = 0.678	*p* = 0.094
	p62	1 ± 0.16	1.19 ± 0.34	1.05 ± 0.09	1.67 ± 1.10	*p* = 0.090	*p* = 0.262	*p* = 0.321
	MAO-A	1 ± 0.21	0.94 ± 0.25	0.69 ± 0.10	1.00 ± 0.36	*p* = 0.332	*p* = 0.162	*p* = 0.070
	MAO-B	1 ± 0.23	0.89 ± 0.13	0.79 ± 0.15	0.94 ± 0.48	*p* = 0.949	*p* = 0.422	*p* = 0.245
	MMP9	1 ± 0.61	0.63 ± 0.36	0.37 ± 0.22	0.50 ± 0.49	*p* = 0.405	*p* = 0.043	*p* = 0.205
Inflammation	CD 68	1 ± 0.24	1.40 ± 0.49	1.59 ± 0.93	4.61 ± 5.41	*p* = 0.145	*p* = 0.086	*p* = 0.203
	TNFα	1 ± 0.81	1.16 ± 1.09	1.24 ± 0.40	0.65 ± 0.80	*p* = 0.661	*p* = 0.758	*p* = 0.270
	IL-6	1 ± 0.34	1.18 ± 0.63	0.95 ± 0.34	1.43 ± 1.52	*p* = 0.336	*p* = 0.788	*p* = 0.639
	COX 2	1 ± 1.09	2.55 ± 2.08	1.77 ± 1.21	1.22 ± 2.72	*p* = 0.346	*p* = 0.797	*p* = 0.160
	Socs3	1 ± 1.12	2.32 ± 1.89	0.71 ± 0.28	0.84 ± 0.59	*p* = 0.088	*p* = 0.091	*p* = 0.219
Angiogenesis	VEGFA	1 ± 0.24	1.14 ± 0.21	1.11 ± 0.52	1.13 ± 0.94	*p* = 0.657	*p* = 0.787	*p* = 0.797
Myogenesis/	Ppargc1a	1 ± 1.02	1.74 ± 1.05	1.99 ± 1.42	1.54 ± 1.21	*p* = 0.602	*p* = 0.340	*p* = 0.213
	MyoG	1 ± 0.41	0.92 ± 0.22	0.55 ± 0.16	1.83 ± 1.71	*p* = 0.152	*p* = 0.616	*p* = 0.048
	PAX7	1 ± 0.62	1.03 ± 0.59	1.04 ± 0.71	0.72 ± 0.78	*p* = 0.662	*p* = 0.660	*p* = 0.526

Total number (n) of male (m) and female (f) wild type (WT) or pancreatic ductal adenocarcinoma (Ca) mice with/without HH treatment. Mean values ± SD and ANOVA results are shown.

**Table 7:** Fraction of centronucleated fibers in gastrocnemius and soleus muscles.

	WT	Ca	WTHH	CaHH	ANOVA		
					PDAC	HH	Interaction
Gastrocnemius muscle							
n (m/f)	9 (3/6)	7 (4/3)	11 (8/3)	6 (3/3)	-	-	-
% all fibers	0.52 ± 0.23	0.71 ± 0.29	0.83 ± 0.27	2.17 ± 0.10	*p* = 0.240	*p* < 0.133	*p* = 0.532
Soleus muscle							
n (m/f)	11 (5/6)	8 (5/3)	6 (6/0)	4 (2/2)	-	-	-
% all fibers	0.08 ± 0.08	0.31 ± 0.21	0.63 ± 0.31	0.75 ± 0.55	*p* = 0.475	*p* = 0.059	*p* = 0.815
% I	0.00 ± 0.00	0.51 ± 0.34	0.58 ± 0.36	0.71 ± 0.71	*p* = 0.325	*p* = 0.243	*p* = 0.569
% IIA	0.18 ± 0.18	0.16 ± 0.16	0.00 ± 0.00	0.33 ± 0.33	*p* = 0.443	*p* = 0.975	*p* = 0.395
% IIBX	00.0 ± 0.00	0.00 ± 0.00	2.50 ± 1.60	1.79 ± 1.79	*p* = 0.679	*p* = 0.019	*p* = 0.679
Gastrocnemius and soleus muscle IIBX fibers							
n (m/f)	20 (8/12)	15 (9/6)	17 (14/3)	10 (5/5)	-	-	-
% IIBX	0.23 ± 0.12	0.33 ± 0.16	1.41 ± 0.59	1.58 ± 0.74	*p* = 0.760	*p* = 0.006	*p* = 0.938

Total number (n) of male (m) and female (f) wild type (WT) or pancreatic ductal adenocarcinoma (Ca) mice with/without HH-treatment. Data are given as mean values ± SD, *p* values by two-factorial ANOVA applied to the total study population are given on the right.

**Table 8:** Pre- and post-synaptic area and overlap of NMJ in quadriceps muscle.

	WT	Ca	WTHH	CaHH	ANOVA		
					PDAC	HH	Interaction
n (m/f)	5 (3/2)	4 (4/0)	5 (3/2)	5 (3/2)	-	-	-
BTX + area (µm^2^)	2.85 ± 0.18	3.06 ± 0.40	2.55 ± 0.14	2.17 ± 0.10	*p* = 0.680	*p* = 0.013	*p* = 0.182
vAChT + area (µm^2^)	3.12 ± 0.28	4.31 ± 0.58	3.65 ± 0.27	2.73 ± 0.09	*p* = 0.683	*p* = 0.124	*p* = 0.005
vAChT/BTX overlap (µm^2^)	0.53 ± 0.03	0.98 ± 0.25	0.68 ± 0.09	0.48 ± 0.03	*p* = 0.279	*p* = 0.127	*p* = 0.010
vAChT/BTX overlap (%)	18.61 ± 0.57	30.54 ± 4.20	26.40 ± 2.34	21.99 ± 1.60	*p* = 0.125	*p* = 0.872	*p* = 0.003

Total number (n) of male (m) and female (f) wild type (WT) or pancreatic ductal adenocarcinoma (Ca) mice with/without HH treatment. Data are given as mean values ± SD, BTX = α-Bungarotoxin, vAChT = vesicular acetylcholine transporter. *p*-values by two-factorial ANOVA applied to the total study population are given on the right.

## Data Availability

The data sets used and/or analyzed during the current study are available from the corresponding author on reasonable request.

## Abbreviations

ANOVA	analysis of variance
BAX	Bcl2-associated X protein
Bcl2	B-cell lymphoma 2
BTX	α-bungarotoxin
Ca	untreated 3xtransgenic mice with PDAC
CaHH	HH-treated 3xtransgenic mice with PDAC
COX2	cyclooxygenase 2 (syn: Prostaglandin endoperoxide synthase 2 (PTGS2))
CSA	cross-sectional area (of muscle fibers in transversal cryosections)
DYRK1 A/B	dual-specificity tyrosine phosphorylation regulated kinases 1 A/B
FBXO32	F-box only protein 32 (syn: MAFbx, Atrogin1)
GSSG	glutathione disulfide
rGSH	reduced glutathione
tGSH	total glutathione
HH	Harmine-Hydrochloride
IL	interleukin
KPC	denotes the transgenic mouse model of PDAC, that incorporates mutant
	endogenous alleles of the **K**ras and **P**53 genes using **C**re-Lox technology
	according to Hingorani et al. [44]
MAO-A/B	monoamino oxidase A/B
MMP9	matrix metallopeptidase 9
MuRF1	muscle ring finger protein 1 (syn: TRIM63)
NMJ	neuromuscular junction (motor endplate)
PDAC	pancreatic ductal adenocarcinoma
p62	ubiquitin-binding protein p62 (syn: Sequestosome 1 (Sqstml1))
PAX7	paired box protein 7
Ppargc1a	peroxisome proliferator-activated receptor gamma coactivator 1-alpha
	(syn: PGC-1α)
RER1	retention of endoplasmatic reticulum 1 protein
ROS	reactive oxygen species
RT-qPCR	real-time quantitative polymerase chain reaction
Socs3	suppressor of cytokine signaling 3
TEM	transmission electron microscopy
TNFα	tumor necrosis factor α
WT	untreated wild type mice
WTHH	HH-treated wild type mice
vAChT	vesicular acetylcholine transporter (syn: SLC18A3)
VEGFA	vascular endothelial growth factor A
